# Supplementary material for: Emergent trees in Colophospermum mopane woodland: influence of elephant density on persistence versus attrition
Source: PeerJ. 2024 Feb 26;12:e16961. doi: 10.7717/peerj.16961 (PMC10903334; doi:10.7717/peerj.16961)
Supplement: Table S1 [file peerj-12-16961-s001.docx]

| Factor | df | Sum of squares | Variation explained (%) | F | *P* |
| --- | --- | --- | --- | --- | --- |
| Null |  | 59.7 |  |  |  |
| Height layer | 3 | 43.3 | 48.9 | 16.8 | 0.0001 |
| Elephant density | 2 | 40.2 | 9.1 | 4.7 | 0.012 |
| Interaction | 6 | 26.1 | 42.0 | 7.2 | 0.0001 |
