# Supplementary material for: Emergent trees in Colophospermum mopane woodland: influence of elephant density on persistence versus attrition
Source: PeerJ. 2024 Feb 26;12:e16961. doi: 10.7717/peerj.16961 (PMC10903334; doi:10.7717/peerj.16961)
Supplement: Table S2 [file peerj-12-16961-s002.docx]

|  |  | OBE |  |  | NBE |  |  | OBU |  |  |
| --- | --- | --- | --- | --- | --- | --- | --- | --- | --- | --- |
| Factor | df | *r^2^* | F | *P* | *r^2^* | F | *P* | *r^2^* | F | *P* |
| Height | 1 | 0.44 | 28.78 | 0.0001 | 0.34 | 5.13 | 0.029 | 0.17 | 15.13 | 0.0001 |
| Elephant density | 2 | 0.26 | 8.45 | 0.0001 | 0.42 | 3.15 | 0.053 | 0.69 | 30.15 | 0.0001 |
| Interaction | 2 | 0.30 | 9.98 | 0.0001 | 0.24 | 1.83 | 0.874 | 0.14 | 6.20 | 0.01 |
