# Supplementary material for: Emergent trees in Colophospermum mopane woodland: influence of elephant density on persistence versus attrition
Source: PeerJ. 2024 Feb 26;12:e16961. doi: 10.7717/peerj.16961 (PMC10903334; doi:10.7717/peerj.16961)
Supplement: Table S3 — Analysis of variance table for the influence of year and height layer on changes in density (loge transformed data) and canopy volume (log10-transformed data) of riverine mopane woodland, Gonarezhou National Park, between 2014 and 2022 [file peerj-12-16961-s003.docx]

| Factor | df | Sum of squares | F | *P* |
| --- | --- | --- | --- | --- |
| *Density* |  |  |  |  |
| Year | 1 | 0.06 | 0.0714 | 0.7902 |
| Height class | 4 | 438.31 | 128.3346 | <2e-16 |
| *Canopy volume* |  |  |  |  |
| Year | 1 | 0.035 | 0.1311 | 0.7185 |
| Height class | 4 | 131.484 | 123.1129 | <2e-16 |
| *Canopy volume* |  |  |  |  |
| Year | 1 | 0.0928 | 0.7973 | 0.3752 |
| Height layer | 4 | 5.8966 | 12.6704 | 1.186e-07 |
