# Supplementary figures and images for: Emergent trees in Colophospermum mopane woodland: influence of elephant density on persistence versus attrition
Source: PeerJ. 2024 Feb 26;12:e16961. doi: 10.7717/peerj.16961 (PMC10903334; doi:10.7717/peerj.16961)

|                                                                                        |                                                                                        |                                                                                         |                                                                                          |
|----------------------------------------------------------------------------------------|----------------------------------------------------------------------------------------|-----------------------------------------------------------------------------------------|------------------------------------------------------------------------------------------|
| A<br>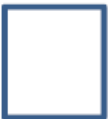 | B<br>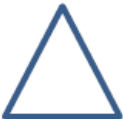 | C<br>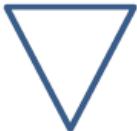 | G<br>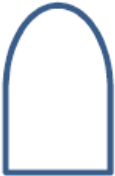 |
| D<br>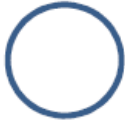 | E<br>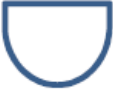 | F<br>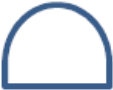 |                                                                                          |

Supplement: Figure S1 — Seven basic shapes of a woody plant canopy according to Melville, Cauldwell & Bothma (1999). [file peerj-12-16961-s017.pdf]

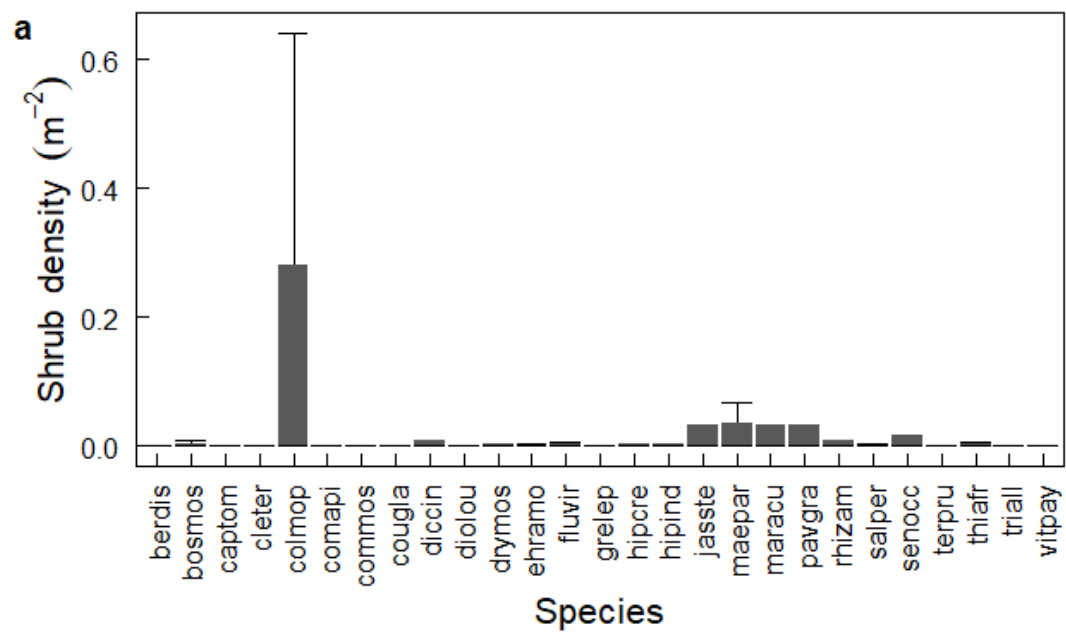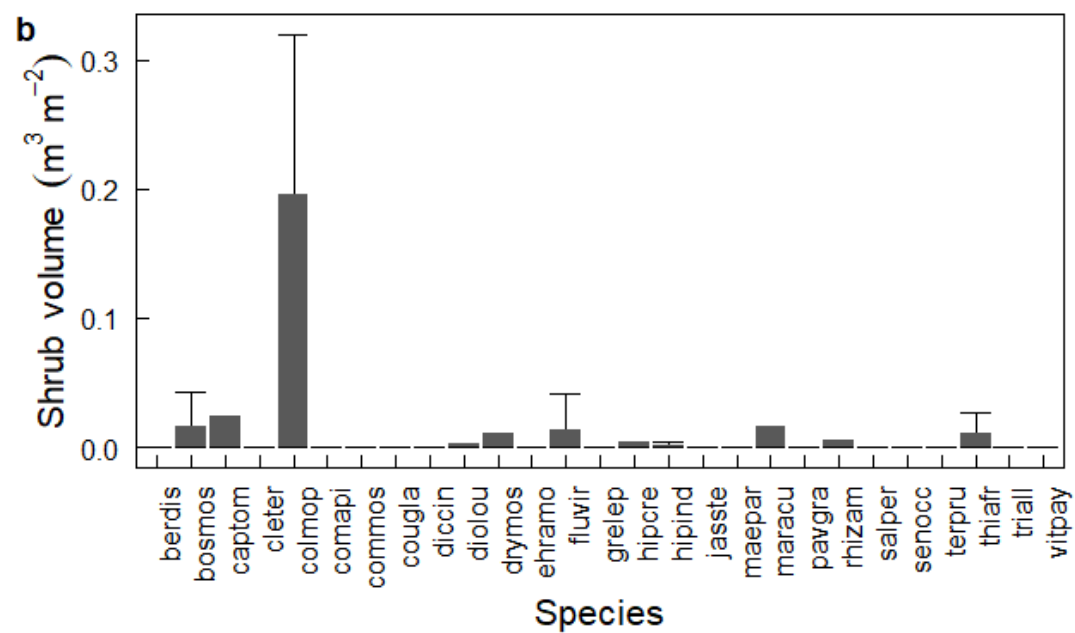

Supplement: Figure S2 — Average shrub (a) density, and (b) canopy volume (n = 7) of 29 shrub species encountered. Key to species: berdis, Berchemia discolor; bosmos, Boscia mossambicensis; captom, Capparis tomentosa; cleter, Clerodendrum ternatum; colmop, Colophospermum mopane; comapi, Combretum apiculatum; commos, Combretum mossambicense; cougla, Maerua edulis (Courbonia glauca); diccin, Dichrostachys cinerea; diolou, Diospyris loureiriana; drymos, Drypetes mossambicensis; ; ehramo, Ehretia amoena; fluvir, Flueggea virosa; grelep, Grewia lepidopetala; hipcre, Hippocratea crenata; hipind, Hippocraytea indica; jasste, Jasminum stenolobum; maepar, Maerua parvifolia; maraca, Markhamia zanzibarica; pavgra, Pavetta gracillima; rhizam, Rhigozum zambeziacum; salper, Salvadora persica; senocc, Senna occidentalis; terpru, Terminalia prunioides; thiafr, Thilachium africanum; triall, Empogona kirkii (Tricalysia allenii); vitpay, Vitex payos. [file peerj-12-16961-s018.pdf]

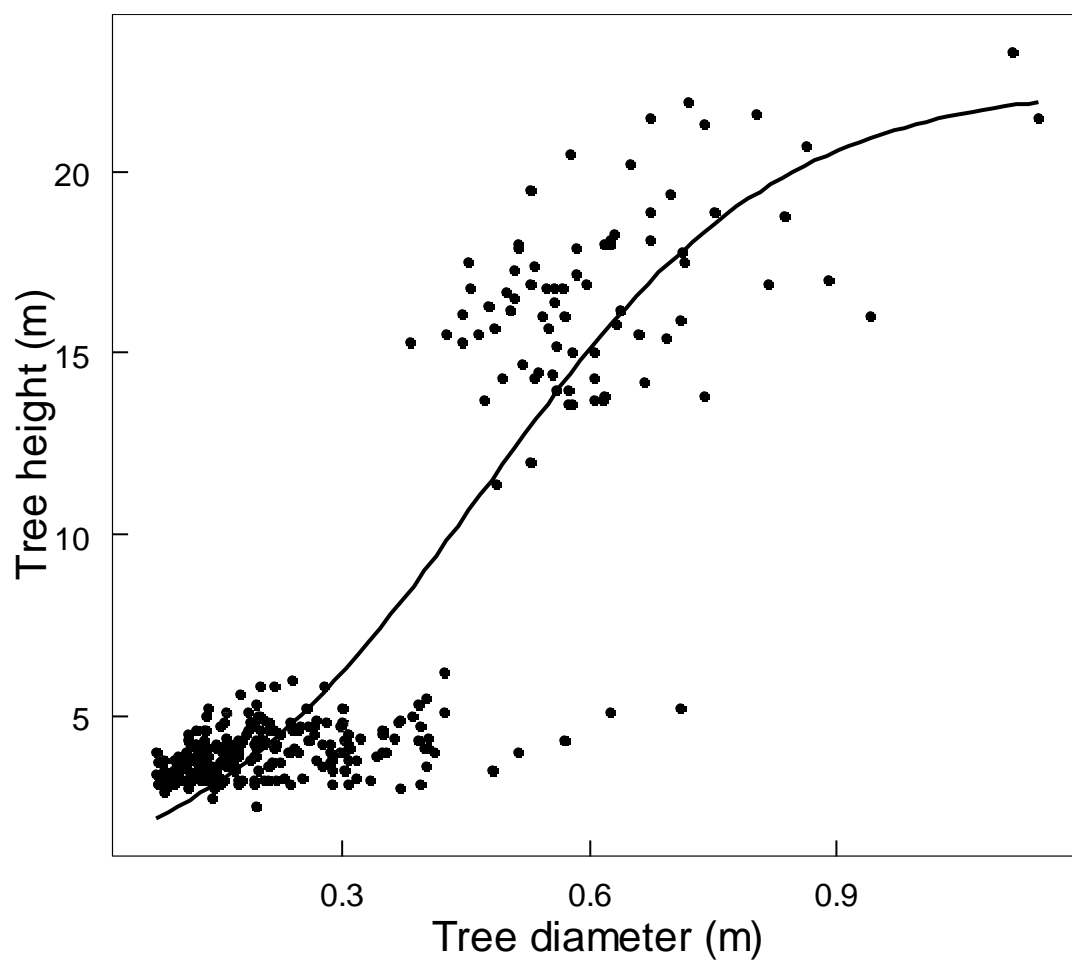

Supplement: Figure S3 — Tree height in relation to the diameter of the main stem (n = 312; only stems with a circumference of >20 cm) is described by a logistic relationship y = 22.43158/(1 + exp(−0.47110∗(x − 0.17881))) (adjusted R2 = 0.7966). [file peerj-12-16961-s019.pdf]

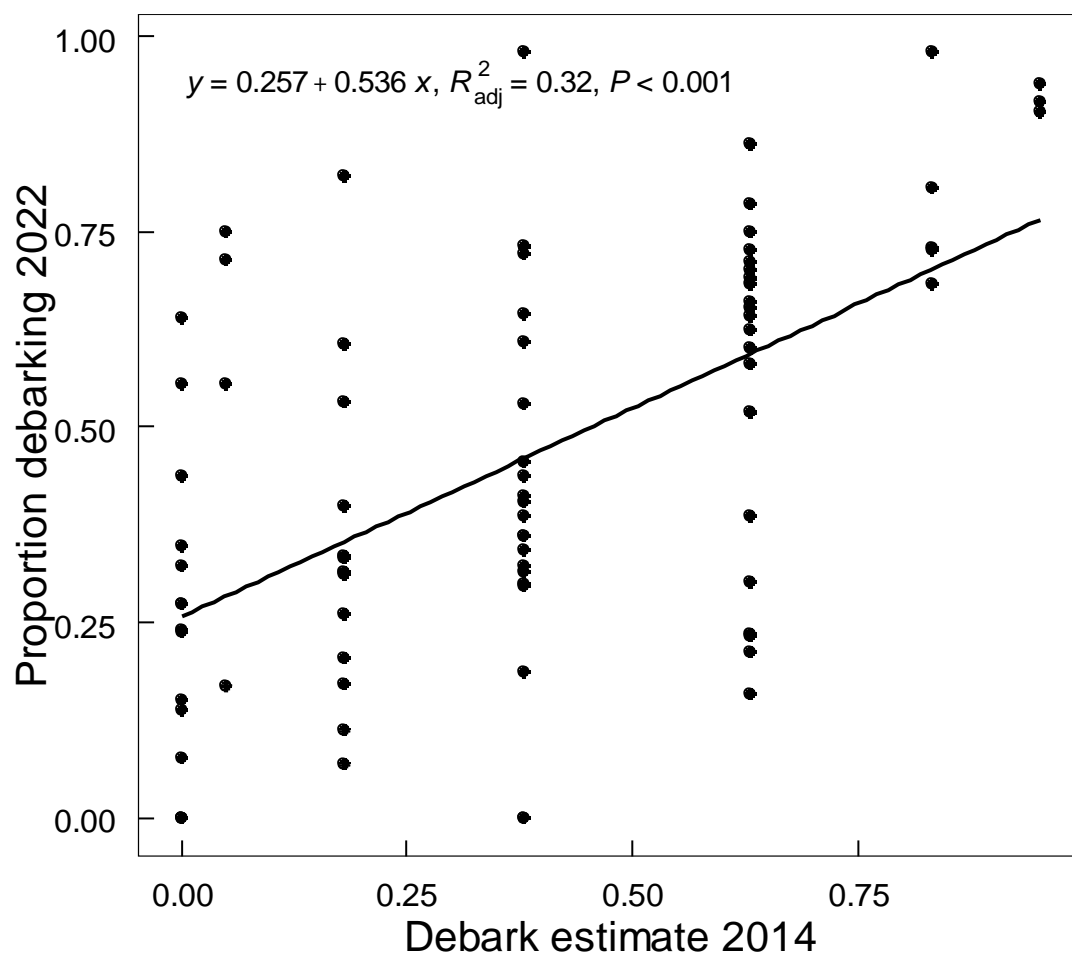

Supplement: Figure S4 — Relationship between the proportion of stem circumference debarked in 2014 (midpoint of a percentage ranking scale) and the proportion debarked of the of the same tree’s stem in 2022 (directly measured), described by a linear relationship y = 0.53565x + 0.25674 ( F1,80 = 39.19, P = 1.7965 e −08; adjusted R2 = 0.3204). [file peerj-12-16961-s020.pdf]
