# Supplementary material for: Emergent trees in Colophospermum mopane woodland: influence of elephant density on persistence versus attrition
Source: PeerJ. 2024 Feb 26;12:e16961. doi: 10.7717/peerj.16961 (PMC10903334; doi:10.7717/peerj.16961)
Supplement: Appendix S1 [file peerj-12-16961-s021.pdf]

Appendix S1. Changes in the reedbeds at Chipinda Pools

This appendix summarises in photographs the changes which have occurred in the abundance of the *Phragmites mauritianus* Kunth reedbeds along 4.2 km of the Runde River at Chipinda Pools, Gonarezhou National Park, since the 1970s. The extent of the reedbeds evident for the 1970s (Figure A1) persisted until the flood resulting from Cyclone Eline in 2000 stripped the reedbeds, with the denuded sandbars still evident in 2006 (Figure A2). These reedbeds had recovered to their zenith by August 2013 (Figure A3), and by October 2015 were being heavily grazed by mainly bull elephants (Figure A4). Heavy grazing by elephants persisted until January 2017 (Figure A5), but the flood resulting from Cyclone Dineo on the 16/17th February 2017 once again stripped the reedbeds (Figure A6) that had not recovered much by September 2022 (Figure A7).

The amount of forage provided by well-developed reedbeds, and the number of elephants they could support, was estimated for the 4.2 km of reedbeds along the Runde River at Chipinda Pools. The area covered by reedbeds, measured directly from a Google Earth image of May 2013, was 69 ha. An estimate of the standing crop of *Phragmites mauritianus* was not found but that of the morphologically very similar *Phragmites australis* (Cav.) Steud is approximately 3 kg m^{-2} (Tarr et al. 2004; Murray-Hudson and Mmopelwa 2011). Thus the Chipinda Pools area supported 2070 tonnes of reeds (30 tonnes ha^{-1}). Assuming only 50 % of above-ground biomass was available (1035 tonnes), and that an adult bull (6000 kg) consumes 1.5 % (Owen-Smith, 1992) of its body weight per day (90 kg), these reedbeds can support about 11500 elephant days of foraging.

Other reedbeds along the river have experienced a similar history.

References

Murray-Hudson M, Mmopelwa G 2011. Biomass production and economic value of *Phragmites communis* reedbeds in the southern Okavango Delta, Botswana. African Journal of Plant Science and Biotechnology 5, 16-20.

Owen-Smith RN 1992. Megaherbivores. The influence of very large body size on ecology. Cambridge University Press, Cambridge.

Tarr JA, van Rooyen MW, Bothma JdP 2004. The response of *Phragmites australis* to harvesting pressure in the Muzi Swamp of the Tembe Elephant Park, South Africa. Land Degradation and Development 15, 487-497.

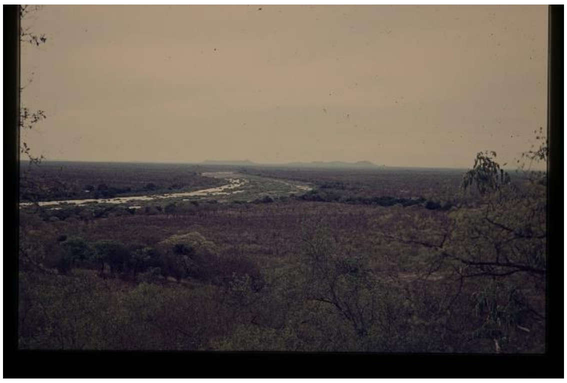

Figure A1. Copyright Angus Anthony. Taken in 1975/6, the photograph shows the extent of reedbed cover of the Runde River at Chipinda Pools, Gonarezhou National Park.

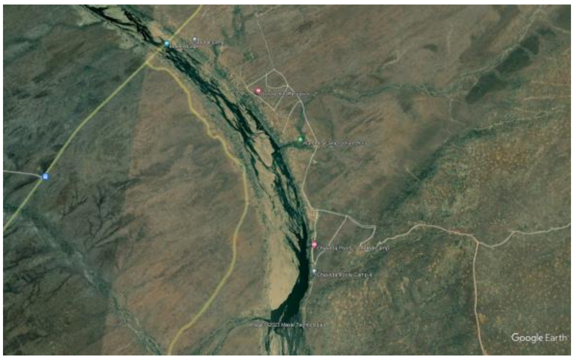

Figure A2. A Google Earth image taken on the 3rd November, 2006, six years after Cyclone Eline. The image shows the denuded sand bars that once supported reed beds along 4.2 km of the Runde River, at Chipinda Pools, Gonarezhou National Park.

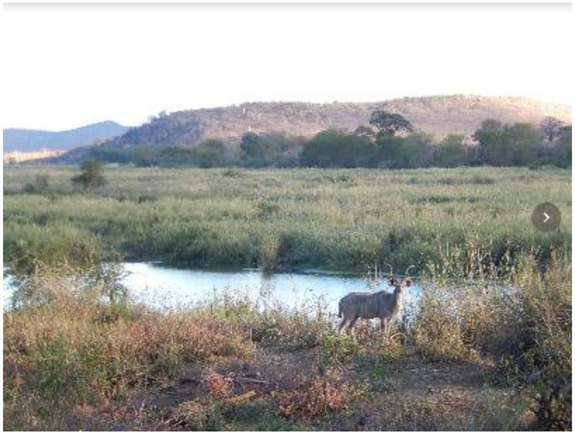

Figure A3. Copyright E. van der Westhuizen. The reedbeds of *Phragmites mauritianus* at Chipinda Pools in August 2013, having recovered to their zenith.

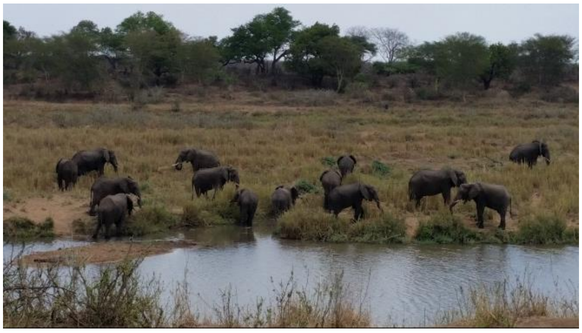

Figure A4. Copyright E. van der Westhuizen. Elephants grazing on the reedbeds of *Phragmites mauritianus* at the height of the dry season in October 2015. Elephant grazing was estimated to have reduced the biomass of the reedbeds by at least 40% compared with the biomass shown in Figure 3 (the effect of other grazers was negligible).

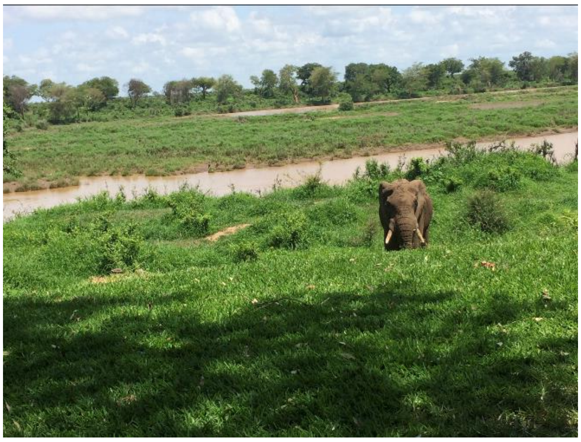

Figure A5. Copyright E. van der Westhuizen. Taken in January 2017, the reedbeds (beyond the elephant, which is on a grassed bank) are well grazed but cover most of the sandbars of the Runde River at Chipinda Pools.

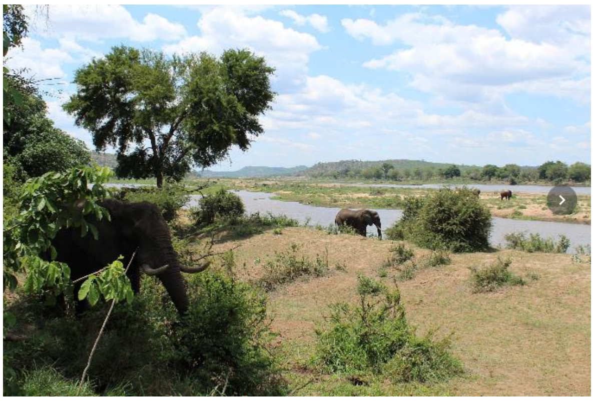

Figure A6. Copyright E. van der Westhuizen. The state of the sandbars in December 2017, following the flood resulting from Cyclone Dineo in February 2017. The reedbeds have been stripped – nearly all the greenery evident is alien herbaceous plants.

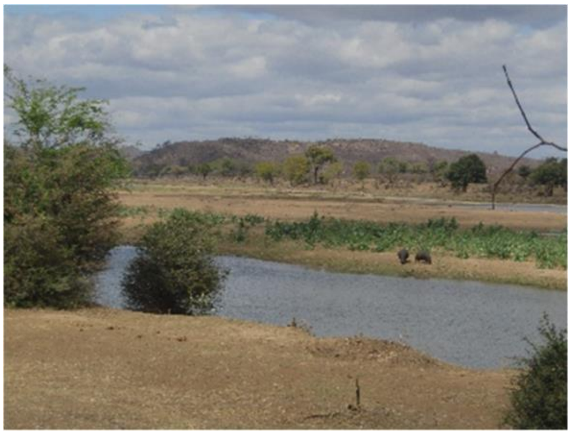

Figure A7. Copyright T. O'Connor. The reedbeds had not recovered much by September 2022; unbrowsed alien herbaceous plants remain evident.
